# The Use of *Saccharomyces cerevisiae* Supplemented with Intracellular Magnesium Ions by Means of Pulsed Electric Field (PEF) in the Process of Bread Production

**DOI:** 10.3390/foods11213496

**Published:** 2022-11-03

**Authors:** Urszula Pankiewicz, Ewelina Zielińska, Aldona Sobota, Anna Wirkijowska

**Affiliations:** 1 Department of Analysis and Food Quality Assessment, University of Life Sciences in Lublin, Skromna 8 Street, 20-704 Lublin, Poland; 2 Department of Plant Food Technology and Gastronomy, University of Life Sciences in Lublin, Skromna 8 Street, 20-704 Lublin, Poland

**Keywords:** bread, magnesium, PEF, antioxidant properties

## Abstract

Bread was supplemented with magnesium through an addition of yeasts subjected to the effect of PEF at optimised parameters to obtain the maximum bioaccumulation of magnesium in cells. Bread produced with the use of yeasts supplemented with magnesium by means of PEF was characterised by its highest content, at 39.3 mg/100 g, which was higher by 50% and 24%, respectively, compared to the control bread sample with an admixture of yeasts cultured without any addition of magnesium and with no PEF treatment and to the control bread sample with an admixture of yeasts cultured with an addition of magnesium but no PEF treatment. The addition of yeasts supplemented with magnesium using PEF in bread production did not cause any statistically significant changes in the chemical composition of any of the analysed samples. However, statistically significant changes were noted in the technological properties of breads produced with an admixture of yeasts supplemented with magnesium by means of PEF treatment. An increase of moisture to 54.03 ± 0.29% led to a reduction of the total baking loss. No statistically significant differences were noted in the bread volume in samples K1, K2, and P, varying from 239 to 269 cm^3^/100 g.

## 1. Introduction

The phenomenon of the deficit of micro- and macroelements, e.g., magnesium, is an important problem facing humanity [1]. Magnesium plays an important role in the correct functioning of the human organism. It has an impact on the functioning of the heart, brain, and skeletal muscles. The element is a cofactor of enzymatic reactions, participates in the metabolism of carbohydrates, and enhances synaptic plasticity, affecting the process of learning and memorising [2]. A study conducted on the American population revealed that 60% of Americans are not supplied magnesium in amounts sufficient for the human organism. Magnesium deficit increases the risk of cardiac diseases, hypertension, nephrolithiasis, and depressive disorders [3,4].

Food supplementation with micro- and macroelements is a highly effective and inexpensive strategy for the improvement of the health status of the population. It is one of the most profitable methods of preventing microelement deficiencies in humans that result from the improper diet [1]. The supply of metal ions in the form of easily assimilable protein complexes results in a high assimilability of ions accumulated in microbial cells, e.g., *Saccharomyces cerevisiae* [5,6,7,8]. Yeast binds metal ions from the environment and then integrates them into cellular structures. In this way, permanent complexes with proteins, so-called bioplexes or metal proteins, are formed [9]. Bioelements in the form of metalloprotein are better absorbed by the body in comparison with pharmaceutic preparations based on organic or inorganic salts of these elements. Magnesium bound in the form of protein complexes in cells of microorganisms with an assistance of PEF is absorbed in the small intestine in a manner analogous to proteins and peptides [10].

The application of pulsed electric field (PEF) contributes to an intensification of more-easily absorbed magnesium accumulation in cells of the yeast *S. cerevisiae* [11]. The field’s effect consists of the induction of short electric pulses within a specific time period [12]. Pulsed electric field has a direct impact on the permeability of cellular membranes. In the process of reversible electroporation, PEF induces transitional permeability of the membrane, as a result of which structures called “pores” or “nanopores” are formed, which facilitate the exchange of components with the environment of the cell [13]. In such a situation, it is possible to introduce chemical compounds, hydrophilic medicines, or large molecules such as DNA into the cytoplasm of the cells. The increased permeability of the cellular membrane can persist from several seconds to as long as several hours after applying PEF [14]. Suitable optimised parameters of PEF (field intensity, pulse duration, number of applied pulses and their frequency) [15] can produce specific effects [16]. 

All over the world, bread is one of the most widely consumed food products. Bread plays an essential role in nutrition, resulting from the proper balance of macronutrients in its composition; in addition, it provides some micronutrients and minerals. It is an important source of energy and a significant source of protein, complex carbohydrates (mainly starch), dietary fibre, vitamins (especially B vitamins), and minerals [17]. The consumption of bread is also recommended in all dietary guidelines, with bread and grain products forming the basis of the food pyramid [18]. To replace the loss of nutrients in flour that occurs during wheat processing and reduce the risk of deficiency in the body, the baking industry has enriched white bread with various nutrients, such as iron, magnesium, thiamine, riboflavin, and niacin [19,20]. Magnesium (Mg) is involved in the enzyme reactions of carbohydrate, protein, and energy metabolism and in maintaining body tissue’s structural and functional integrity. Unfortunately, given the food choices in many households worldwide, this daily intake is rarely met. Fortification of cereal products with magnesium is therefore justified as a popular food item. Another good example of a product enriched with magnesium is cheese [21].

The choice of a bakery product with an innovative recipe produced with an addition of yeasts supplemented with magnesium using PEF resulted in meeting the requirements of present-day consumers, who are increasingly interested in improved diet and health. The aim of this study was to use *S. cerevisiae* yeast enriched with intracellular magnesium ions using PEF for bread production and to analyse the effect of this addition on bread quality. Magnesium-enriched bread can provide an additional source of this element in the diet.

Many authors, including Skibniewska et al. [22], Capar and Cunningham [23], and Rybicka et al. [24], determined the magnesium content in bread and found that it was, respectively, 8.64 mg/100 g, 22.3 mg/100 g, and 14 mg/100 g. The aim of our research was to increase the magnesium content in bread by adding yeast with a higher accumulation of this element caused by the treatment of cells with PEF.

## 2. Materials and Methods

### 2.1. Culture Maintenance and Inoculum Preparation

*Saccharomyces cerevisiae* 11 B1 (industrial strain) from the Yeast Plant (Lublin, Poland) was used (K1—yeast not treated with PEF and without magnesium in the medium; K2—yeast not treated with PEF and with magnesium in the medium 100 µg Mg/mL; P—yeast treated with PEF and with magnesium in the medium 100 µg Mg/mL). Medium for agar slants and inoculum growth were according to the procedure described by Pankiewicz and Jamroz and Blackwell et al. [11,25]. The *S. cerevisiae* yeast marked K is a dry yeast that was purchased in a local store.

### 2.2. Biomass Cultivation under Optimised Conditions

Biomass cultivation under optimised conditions was performed according to the method described previously by Pankiewicz and Jamroz [11].

### 2.3. PEF Treatment and Enrichment with Magnesium

*S. cerevisiae* cultures grown in flasks were agitated under optimised conditions [11], that is, on 15 min exposure of the 20 h grown culture to PEF (electroporator ECM 830, BTX Harvard Apparatus, Hollistone, MA, USA) of the 2000 V and 20 µs pulse width; at the field frequency of 1 Hz, accumulation of magnesium in the yeast biomass reached maximum 3.98 mg/g dm. Samples not treated with PEF and without magnesium in the medium (K1) or with 100 µg Mg/mL (K2) served as controls.

### 2.4. Bread-Making Procedure

The dough was prepared using the single-stage method [26]. All doughs were prepared according to the same recipe: wheat flour, 3% w.w. of yeast (moisture 68%) (K—dry yeast bought in local store; K1—sample not treated with PEF and without magnesium in the medium; K2—samples not treated with PEF and with magnesium in the medium 100 µg Mg/mL; P—samples treated with PEF and with magnesium in the medium 100 µg Mg/mL), 1% salt, and 56.7% water per amount of flour used. The dough underwent 60 min fermentation (Tefi Klima pro 100; DEBAG Deutsche Backofenbau GmbH, Bautzen, Germany; temperature 28 °C, 85 ± 2% relative humidity), punching (1 min of mixing at low speed), and another fermentation (temp. = 30 °C, t = 30 min). Next, the dough was hand-divided into pieces 290 g ± 5 g, formed, placed in pans, and left to proof in the fermentation chamber (temp. = 30 °C, t = 30 min). Baking was carried out in a laboratory oven, Helios 4060/3 PRO (Debag Deutsche Backofenbau GmbH, Bautzen, Germany), at 230 °C for 30 min. Three loaves of bread were baked for each variant of yeast used. After baking, the bread was cooled at ambient temperature for 2 h, weighed, and placed in polyethylene bags. The samples were stored for 24 h at room conditions (24 °C, 50% RH) for quality assessment.

### 2.5. Evaluation of Bread Quality Characteristics

Evaluation of bread quality included determination of bread yield according to Ambrosewicz-Walacik et al. [27], total baking loss according to Bakare et al. [28], moisture content of crumb (AACC, 2000; Method 44–15.02) [29], and specific volume of bread according to Wirkijowska et al. [26,30].

### 2.6. Determination of Magnesium Concentration in Yeast Cells and Bread

The concentration of magnesium ions in yeast biomass and bread mineralisations was determined using the method of flame atomic absorption spectrophotometry (FAAS, Solaar 939, Unicam) according to Jorhem and Engman [31].

### 2.7. Chemical Analysis of Bread

All measurements were carried out in triplicate. The dry matters of bread were determined by drying samples at 130 ± 1 °C for 3 h [29]. Fat content was determined according to AOAC [29] by applying extraction in a Soxhlet apparatus (Tecator Soxtec System HT 1043 extraction unit, Gemini, Apeldoorn, The Netherlands), and protein content was assayed according to Kjeldahl method, with a nitrogen-to-protein conversion factor of 6.25 [29]. Ash content was analysed according to PN-A-79011-8 (1998). Carbohydrates were calculated as the difference between 100% and the sum of the percentages of water, protein, total lipid (fat), and ash [32].

### 2.8. Antioxidant Properties

#### 2.8.1. Extraction of Bioactive Compounds

Samples (1 g) of the bread were ground and extracted with 10 mL of 4:1 ethanol/water (*v/v*) for 120 min in a laboratory shaker. Next, the samples were centrifuged at 3000 g for 10 min. The supernatant was stored at –20 °C for further analysis [33].

#### 2.8.2. DPPH (2,2-Difenylo-1-pikrylohydrazyl) Radical Scavenging Activity

The DPPH^•^ scavenging activity was measured by the modified method of Brand-Williams et al. [34]. A 0.1 mL sample volume was mixed with 0.9 mL of a 6 µM solution of DPPH^•^ in 75% methanol. The absorbance was read at 515 nm after 30 min of the reaction.

Scavenging activity (%) = [1 − (A sample/A control)] × 100(1)

where A sample is the absorbance of the mixture of sample and DPPH^•^; A control is the absorbance of the control (DPPH^•^ solution).

The results were expressed as Trolox equivalent antioxidant activity (TEAC) values (mM Trolox/g sample).

#### 2.8.3. ABTS (3-Etylobenzotiazolino-6-sulfonianu) Radical Scavenging Activity

The ABTS^•+^ assay was measured by the modified method of Re et al. [35]. A 0.05 mL amount of each sample was mixed with 2.95 mL of the ABTS^•+^ solution. The absorbance was read at 734 nm after 30 min of the reaction.

Scavenging activity (%) = [1 − (A sample/A control)] × 100(2)

where A sample is the absorbance of the mixture of sample and ABTS^•+^; A control is the absorbance of the control (ABTS^•+^ solution).

The results were expressed as Trolox equivalent antioxidant activity (TEAC) values (mM Trolox/g sample).

### 2.9. Statistical Analysis

All determinations were made in triplicate. Significant differences between individual groups were found using the Student’s *t*-test at the level of significance α = 0.05. Statistical processing of results was carried out using the R version 3.1.2 (GNU General Public License, Boston, MA, USA).

## 3. Results and Discussion

### 3.1. Determination of Magnesium Concentration in Saccharomyces cerevisiae and Bread

It is common knowledge that magnesium is an essential element for the correct growth and development of all living organisms. Metal bonding by *S. cerevisiae* is an effective, fast, and cheap process; ions are most frequently bound to the cell wall through biosorption [8]. To enhance the effectiveness of the process, pulsed electric field was applied, using parameters optimised in an earlier study (Section 2.3 PEF treatment and enrichment with magnesium) [11].

PEF treatment intensified the accumulation of magnesium ions in cells of *S. cerevisiae*. The accumulation of ions in yeast cultured at an optimum concentration of magnesium in the culturing medium and optimum PEF parameters (sample P) was 4.72 mg/g DM, and it was by 216% and 400%, respectively, relative to yeasts from the control sample K2 (supplemented with magnesium but without PEF treatment) and to yeasts from the control sample K1 (with no magnesium supplementation and no PEF treatment) (Table 1). The magnesium content in bread was from 26.182 mg/100 g to 39.3 mg/100 g (Table 1). Bread produced with the use of yeasts supplemented with magnesium by means of pulsed electric field (P) was characterised by the highest magnesium content, at 39.3 mg/100 g, which was higher by 50% and 24%, respectively, compared to the control sample of bread K1, with addition of yeasts cultured with no magnesium supplementation and no PEF treatment, and to the control sample of bread K2, with addition of yeasts cultured with magnesium supplementation but no PEF treatment. Grembecka et al. [36], in their study on the content of magnesium, phosphorus, zinc, and iron in various kinds of bread, demonstrated that in samples of white bread, the content of magnesium varied from 16.8 to 31.2 mg/100 g, with a mean value of 21 mg/100 g of product. The highest magnesium concentration (31.2 mg/100 g) was noted in toast bread, and the lowest was in Wrocław rolls (16.8 mg/100 g). According to Hussein and Bruggeman [37], the average level of magnesium in bread is 27 mg/100 g, while Skibniewska et al. [22] reported the average magnesium concentration in white bread at the level of 8.64 mg/100 g and Capar and Cunningham [23] at the level of 22.3 mg/100 g. Rybicka et al. [24] demonstrated that the content of Mg for control bread was 14 mg in 100 g, whereas it was higher by 23–31 mg for teff, amaranth, and quinoa. Huang et al. [38] demonstrated magnesium content of 40.51 mg/100 g dry weight of wheat bread. Carocho et al. [39], analysing various kinds of bread, obtained magnesium levels in the range of 28–56 mg/100 g. Wronkowska et al. [40] demonstrated magnesium content in wheat bread at the level of 20 mg/100 g. Most of the authors mentioned [36,39,41,42] obtained a much lower magnesium content in their breads compared to our bread produced with the addition of yeast enriched with magnesium under the PEF conditions.

### 3.2. Bread Quality

The addition of yeasts supplemented with magnesium by PEF in bread production did not cause any statistically significant changes in the chemical composition of all the analysed samples. Detailed results of the analysis are presented in Table 2. Fat content varied within the range from 1.492 to 1.514%, protein content from 10.20 to 11.07%, ash content from 1.019 to 1.020%, and the content of carbohydrates varied from 59.573 to 61.158%.

The quality characteristics of bread are related to the raw materials used in its production, both the basic ones and those added in small amounts [41]. Table 3 presents the basic quality characteristics of bread as affected by the addition of yeasts supplemented with magnesium by means of pulsed electric field treatment.

Crumb moisture of the control bread samples K, K1, and K2 did not differ statistically significantly at the level of 42.49% in the case of sample K, in which magnesium concentration was 28.84 ± 0.30 mg/100 g of bread, and 42.88% in the case of bread K2, in which the concentration of magnesium was at the level of 31.4 ± 0.71 mg/100 g. Bread with addition of yeasts supplemented with magnesium using the method of electroporation (P) was characterised by the highest crumb moisture (54.03%). Wirkijowska et al. [26] emphasised that higher water retention in the crumb is related to a lower loss of moisture content after baking. Higher moisture retention in bread indicates its quality and is directly correlated with the shelf life of bakery products [43]. At the same time, it should be stressed that the bread sample with yeasts supplemented with magnesium using the method of electroporation was characterised by the lowest baking losses (BL and TBL) and statistically higher bread yield (*p* < 0.05) compared to the other three control samples of bread (K, K1, and K2).

Litwinek et al. [44] demonstrated in their study that on the day of baking, the highest crumb moisture was characteristic of rye bread (49–50%). Those authors noted a slightly lower level of crumb moisture in wheat bread both from spelt wheat flour and from common wheat flour. The average crumb moisture of those breads was approx. 47.5% [44]. Even lower levels of crumb moisture (39.6%) were noted in wheat bread by Wirkijowska et al. [26]. Huang et al. [38] assayed wheat bread crumb moisture at the level of 50.78%.

**Figure 1:**
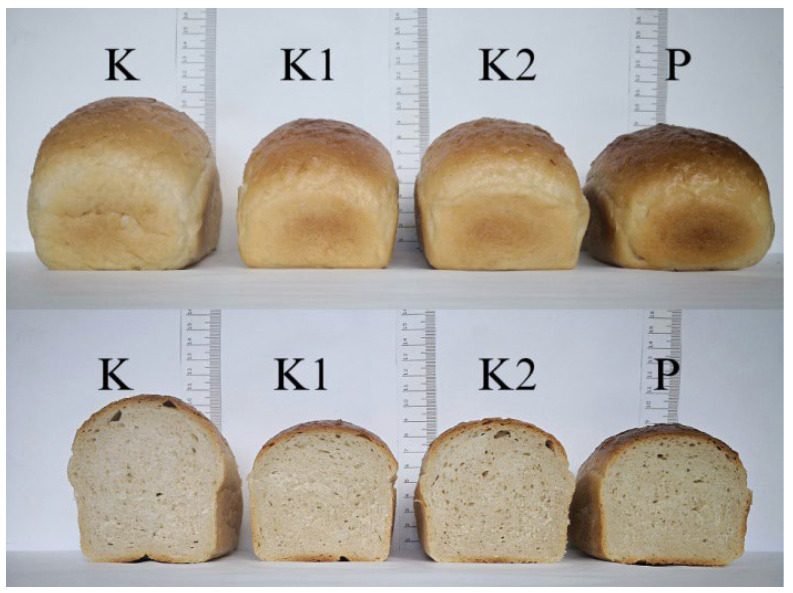
Photographs of bread with *S. cerevisiae* supplemented with magnesium ions. Control samples of the bread with yeast: K, dry yeast; K1, without magnesium and PEF treatment; K2, with magnesium and without PEF treatment; P, with magnesium and PEF treatment.

The loss of moisture content during baking was positively correlated with baking loss (BL and TBL). The total baking loss of the analysed breads varied in the range of 9–11.57%. The highest value of total baking loss (11.57%) was noted for the control sample with an addition of dry yeasts; that sample did not differ statistically significantly from the control samples K1 (with yeasts without magnesium supplementation and with no PEF treatment) and K2 (with yeasts with supplementation with magnesium and with PEF) (Table 3). The lowest level of total baking loss (9.3426%) was noted in bread produced with yeasts supplemented with magnesium by means of PEF treatment. It was lower by 16% and 20%, respectively, compared to the control samples K1 and K. The addition of yeasts supplemented with magnesium by means of PEF contributed to a statistically significant reduction of total baking loss (Figure 1). Litwinek et al. [44] demonstrated in their study total baking loss values of all analysed breads within the range of 12–15.5%, with the values being independent of the kind of analysed bread.

The specific volume of the analysed bread samples varied within the range of 239–334 cm^3^/100 g. The bread produced using dry yeasts was characterised by a significantly higher specific volume (333.76 cm^3^/100 g) relative to the other bread samples. It was higher by 28% compared to the bread produced with an addition of yeasts supplemented with magnesium by means of PEF. No statistically significant differences were noted in the values of the specific volume of samples K1, K2, and P, which varied within the range from 239 to 269 cm^3^/100 g. The addition of yeasts supplemented with magnesium did not have any impact on the specific volume of bread. Huang et al. [38] obtained the specific volume value of 221 cm^3^/100 g of wheat bread. In their study, Wronkowska et al. [40] obtained specific values of bread in the range from 214 to 276 cm^3^/100 g. Wheat bread was characterised by the highest specific volume, at 276 cm^3^/100 g, while addition of acidic whey concentrate to the dough composition caused a significant decrease of bread specific volume to as low as 214 cm^3^/100 g. In the study by Litwinek et al. [44], the highest specific volume of breads was from Graham common wheat flour at 1024 cm^3^, while rye breads and spelt wheat breads were characterised by a fairly stable loaf volume of approximately 888 cm^3^. The specific volume of our bread was even higher compared to the results obtained by other authors [38,40].

Ranhotra et al. [20] found that fortification of bread by adding 44.1 mg of magnesium per 100 g of flour in the form of dioxide, hydroxide, or carbonate resulted in reduced volume and deterioration of taste. They studied the effects of a range of organic (lactate, acetate, and citrate) and inorganic (chloride, phosphate, oxide, carbonate, sulphate, and hydroxide) sources of magnesium, but especially oxide, on the quality of bread produced by the sponge dough, no-time dough, and continuous mixing procedure. When added at a level of 44.1 mg of magnesium per 100 g of flour, magnesium-rich sources (oxide, hydroxide, and carbonate) significantly raised the pH of the bread and negatively affected loaf volume and overall quality, including taste. Further, studies showed that adding magnesium to the dough or using flour pre-enriched with magnesium significantly improved loaf volume and overall quality when the increase in dough pH was stopped by adding acetic acid.

In many Western countries, bread is an important source of Mg^2+^. Lopez et al. [42] compared the effects of different types of bread fermentation on Mg^2+^ bioavailability in rats. The authors found that although yeast fermentation minimises the adverse effects of phytic acid on Mg^2+^ bioavailability, sourdough bread was a better source of available Mg^2+^. The enrichment of yeast with a bioavailable form of magnesium, especially for wheat bread, seems appropriate and fully justified.

### 3.3. Antioxidant Properties

Diet plays an important role in preventing many chronic diseases, so nowadays, consumers prefer healthier foods. For this reason, the industry and scientists are engaged in optimising bread-production technology to improve quality, taste, functionality, and health-promoting properties [45]. One of them is antioxidant potential, which can prevent the occurrence of oxidative stress and the development of civilisation diseases. Furthermore, bread is an important processed wheat product consumed globally, so its health-promoting properties are more likely to affect the body.

Antioxidant activities of bread extracts were determined based on their scavenging activity of the stable ABTS and DPPH free radicals. The radical scavenging activity ranged from 0.659 ± 0.04 mM TE/g to 0.905 ± 0.05 mM TE/g for ABTS and from 0.184 ± 0.0 mM TE/g to 0.269 ± 0.04 mM TE/g for DPPH. Similar results of antioxidant activity for wheat bread ethanolic extracts were obtained by Peng et al. [46]. As shown in Table 4, the radical scavenging activity of bread with *S. cerevisiae* supplemented with magnesium ions and PEF treatment was significantly higher than in control samples (*p* < 0.05). This is due to the highest magnesium ion content in this bread sample.

The reduced form of metal ions can potentially increase antioxidant activity [47]. Other authors found that the percent inhibition of radical species was almost two-fold higher in metal complexed quercetin molecules (with Mg^2+^ and Ca^2+^) compared to free quercetin molecules. Similarly, the antioxidant activity of catechin was significantly higher in the presence of Ca^2+^ and Mg^2+^ [48]. This phenomenon is observed because metal complexes of quercetin molecules can be more easily oxidised by free radicals than free quercetin molecules [49]. Applying these conclusions to the results of our analysis, we can suggest that the presence of magnesium ions may have a positive effect on the antioxidant activity of antioxidants present in the samples of studied bread.

Additionally, calcium and magnesium ions are important for redox balance and cells’ production of reactive oxygen species. Magnesium deficiency stimulates respiratory chain activity, leading to higher amounts of reactive oxygen species (ROS), so an adequate supply can prevent free radical reactions [50,51].

## 4. Conclusions

Bread produced with the use of yeasts supplemented with magnesium by PEF was characterised by its highest content. The addition of magnesium-enriched yeasts to a dough did not cause any statistically significant changes in the chemical composition of any of the analysed samples. However, statistically significant changes were noted in the technological properties (except the bread volume) of breads produced with an admixture of yeasts supplemented with magnesium by means of PEF treatment. The radical scavenging activity of bread with *S. cerevisiae* supplemented with magnesium ions and PEF treatment was significantly higher than in control samples (*p* < 0.05). Bread obtained with yeast enriched with magnesium can provide its additional source in the diet.

## Figures and Tables

**Table 1:** Magnesium in *S. cerevisiae* cells and bread with *S. cerevisiae* supplemented with magnesium ions. Control samples: K, dry yeast; K1, without magnesium and PEF treatment; K2, with magnesium and without PEF treatment; P, with magnesium and PEF.

Yeast	Magnesium Ions in Yeast (mgg d.m.)	Bread	Magnesium Ions in Bread (mg/100 g of bread)
*S. cerevisiae (dry)* K	0.79 ± 0.017 ^d^	K	28.84 ± 0.30 ^c^
*S. cerevisiae* K1	1.16 ± 0.074 ^c^	K1	26.182 ± 0.16 ^d^
*S. cerevisiae* K2	2.18 ± 0.126 ^b^	K2	31.4 ± 0.71 ^b^
*S. cerevisiae* P	4.72 ± 0.09 ^a^	P	39.3 ± 0.39 ^a^

^a,b,c,d,^ Means in the same column indicated by different letters are significantly different (*p*-value < 0.05).

**Table 2:** Chemical composition (mean ± standard deviation) of the bread with *S. cerevisiae* supplemented with magnesium ions in 100 g of the final product. Control samples of the bread with yeast: K, dry yeast; K1, without magnesium and PEF treatment; K2, with magnesium and without PEF treatment.

Bread	Fat (%)	Protein (%)	Ash (%)	Dry Matter (%)	Carbohydrates (%)
	$\bar{x}\pm \mathrm{SD}$				
**K**	1.505 ± 0.02 ^a^	10.21 ± 0.03 ^a^	1.020 ± 0.00 ^a^	73.26 ± 0.57 ^a^	60.525 ± 0.64 ^a^
**K1**	1.492 ± 0.02 ^a^	10.20 ± 0.05 ^a^	1.020 ± 0.00 ^a^	73.87 ± 0.55 ^a^	61.158 ± 0.30 ^a^
**K2**	1.508 ± 0.11 ^a^	11.07 ± 0.93 ^a^	1.019 ± 0.00 ^a^	73.17 ± 0.29 ^a^	59.573 ± 0.73 ^a^
**P**	1.514 ± 0.11 ^a^	11.02 ± 0.82 ^a^	1.019 ± 0.00 ^a^	73.47 ± 0.21 ^a^	59.917 ± 0.29 ^a^

^a^ Means in the same column indicated by different letters are significantly different (*p*-value < 0.05).

**Table 3:** Quality characteristics of bread with *S. cerevisiae* supplemented with magnesium ions. Control samples of the bread with yeast: K, dry yeast; K1, without magnesium and PEF treatment; K2, with magnesium and without PEF treatment; P, with magnesium and PEF treatment.

Bread Samples	Bread Yield	Baking Loss	Total Baking Loss	Crumb Moisture	Specific Volume
	(%)				(cm^3^ 100 g^−1^)
**K**	142.11 ± 0.7 ^b^	9.74 ± 0.23 ^a^	11.57 ± 0.44 ^a^	42.49 ± 0.35 ^b^	333.76 ± 14.77 ^a^
**K1**	143.03 ± 0.62 ^b^	8.45 ± 0.22 ^b^	10.99 ± 0.38 ^a^	42.63 ± 0.14 ^b^	269.33 ± 13.58 ^b^
**K2**	143.12 ± 1.01 ^b^	8.15 ± 0.21 ^b^	10.94 ± 0.63 ^a^	42.88 ± 0.26 ^b^	240.31 ± 11.65 ^b^
**P**	145.69 ± 0.07 ^a^	7.71 ± 0.30 ^c^	9.34 ± 0.04 ^b^	54.03 ± 0.29 ^a^	239.54 ± 16.13 ^b^

^a,b,c^ Means in the same column indicated by different letters are significantly different (*p*-value < 0.05).

**Table 4:** Antioxidant properties of bread with *S. cerevisiae* supplemented with magnesium ions. Control samples: K, dry yeast; K1, without magnesium and PEF treatment; K2, with magnesium and without PEF treatment; P, with magnesium and PEF treatment.

	Antioxidant Activity TEAC (mM/g)	
Bread	ABTS	DPPH
**K**	0.766 ± 0.08 ^b^	0.184 ± 0.0 ^b^
**K1**	0.659 ± 0.04 ^b^	0.187 ± 0.01 ^b^
**K2**	0.766 ± 0.02 ^b^	0.190 ± 0.01 ^b^
**P**	0.905 ± 0.05 ^a^	0.269 ± 0.04 ^a^

^a,b^ Means in the same column indicated by different letters are significantly different (*p*-value < 0.05).

## Data Availability

The data presented in this study are available on request from the corresponding author.
